# The effect and process evaluations of the national quality improvement programme for palliative care: the study protocol

**DOI:** 10.1186/1472-684X-13-5

**Published:** 2014-02-21

**Authors:** Natasja JH Raijmakers, Jolien M Hofstede, Ellen JM de Nijs, Luc Deliens, Anneke L Francke

**Affiliations:** 1 Netherlands Institute for health services research (NIVEL), PO box 1568, 3500 Utrecht, BN, The Netherlands; 2 Center of expertise Palliative Care, University Medical Center Leiden (LUMC), PO box 9600, 2300 RC Leiden, The Netherlands; 3 End-of-Life Care Research Group, Vrije Universiteit Brussel & Ghent University, Laarbeeklaan 103, Brussels 1090, Belgium; 4 Department of Public and Occupational Health (EMGO+), VU University Medical Centre, PO Box 7057, 1007 MB Amsterdam, The Netherlands

**Keywords:** Palliative care, Terminal care, End-of-life care, Effect evaluation, Process evaluation, Quality improvement, Complex intervention, Implementation, Effectiveness

## Abstract

**Background:**

The nationwide integration of palliative care best practices into general care settings is challenging but important in improving the quality of palliative care. This is why the Dutch National Quality Improvement Programme for Palliative Care has recently been launched. This four-year programme consists of about 70 implementation trajectories of best practices. A large evaluation study has been set up to evaluate this national programme and separate implementation trajectories.

**Methods/Design:**

This paper presents the protocol of the evaluation study consisting of a quantitative effect evaluation and a qualitative process evaluation. The effect evaluation has a pre-test post-test design, with measurements before implementation (month 0) and after implementation (month 9) of a best practice. Patients are eligible if they have a life expectancy of less than six months and/or if they are undergoing palliative treatment and provided they are physically and mentally capable of responding to questionnaires. Bereaved relatives are eligible if they have been involved in the care of a deceased patient who died after a sickbed between six weeks and six months ago. Three types of measurement instruments are used: (1) numerical rating scales for six symptoms (pain, fatigue, breathlessness, obstipation, sadness and anxiety), (2) the Consumer Quality Index Palliative Care - patient version and (3) the version for bereaved relatives.

The process evaluation consists of analysing implementation plans and reports of the implementation, and individual and group interviews with healthcare professionals. This will be done nine to eleven months after the start of the implementation of a best practice.

**Discussion:**

This mixed-method evaluation study gives more insight into the effects of the total programme and the separate implementation trajectories. However, evaluation of large quality improvement programmes is complicated due to changing, non-controlled environments. Therefore, it is important that an effect evaluation is combined with a process evaluation.

**Trial registration:**

NTR-4085

## Background

Death comes to us all. Nowadays, acute deaths due to infectious diseases have largely been replaced by non-sudden deaths, caused by e.g. cancer, cardiovascular diseases and dementia [1]. Therefore, the need for palliative care is increasing. High-quality palliative care is important in improving the quality of life of patients and their families facing the problems associated with a life-threatening disease [2]. Since the 1990s, the Dutch government has consistently invested in the optimization of palliative care, and nowadays many best practices are available in the Netherlands. 'Best practices' are defined in this paper as practices, often developed among small groups of patients and professionals, that contribute to the improvement of palliative care and that are transferrable to other settings. Some examples of best practices in the Netherlands are:

The Dutch version of the Liverpool Care Pathway for the Dying Patient [3]. The pathway was developed to aid members of a multi-disciplinary team in matters relating to continuing or discontinuation of treatments and regarding comfort measures during the last days and hours of a patient's life.

PaTz – a systematic approach to optimize palliative care based on the Gold Standard Framework (http://www.goldstandardsframework.org.uk/). Its elements include collaborative meetings of general practitioners and community nurses, the timely identification of patients in need of palliative care and the drafting of an advance care plan [4].

Informare, a tailored method that enables health care professionals to provide timely information about palliative care to patients and relatives [4].

‘Signal box for nursing assistants’, a tool that enables nursing assistants to identify palliative care needs in their patients and pass this information on to other professionals [4].

These best practices are known for their contribution to high-quality palliative care, although so far most of the knowledge about the effects of these best practices on the quality of palliative care is practice-based rather than evidence-based. At the moment, most evidence is available with regard to the Dutch version of the Liverpool Care Pathway (LCP) for the Dying Patient and regarding PaTz. Research has indicated that use of the Dutch version of the LCP leads to a decrease in the symptom burden for patients in the last days of life, a decrease in the grief burden for relatives and an improvement in the documentation of care [5-7]. Furthermore, a recent review showed that the Gold Standard Framework (the British equivalent of PaTz) improves processes in general practice, co-working and the quality of palliative care. Although the direct impact on patients and carers is unknown at present, the Gold Standard Framework has considerable potential to improve palliative primary care [8].

Although large-scale implementation of such best practices appears to be important for improving palliative care, implementation in general health care settings (e.g. general hospitals, general practices, residential elderly care and home care) is challenging, e.g. because professionals in general health care settings are often not specialized in palliative care and often provide care to a variety of patient categories. Moreover, hospital settings in particular are traditionally strongly focused on cure and life prolongation, which influences how palliative care needs are attended to [9].

### National quality improvement programme for palliative care

The Dutch National Quality Improvement Programme for Palliative Care was launched in 2012 with the aim of promoting the implementation of best practices in general health care settings. This four-year programme receives financial and practical support from ZonMw (The Netherlands Organisation for Health Research and Development) and was commissioned by the Dutch Ministry of Health. The programme’s key objectives are to promote that:

•Patients die at their preferred place.

•Patients and relatives feel they are in control regarding palliative care.

•Patients and relatives see palliative care as being coordinated.

•Patients and relatives feel care to be concordant with their needs, preferences and values.

•Patients and relatives receive care for their needs in the physical, psychosocial and spiritual domains.

This programme enables care organizations to improve their palliative care by implementing one or more of the available best practices. The best practices are pre-selected by an independent committee of experts, professionals and patient representatives, taking account of the available evidence, the usability and the transferability of the best practice to general health care settings. The committee assesses potential new best practices every year to include in the National Quality Improvement Program Palliative Care. The nine best practices that are currently pre-selected for implementation are listed in Additional file 1.

Every year during the programme period (2012–2016), there is a call for applications. Representatives of regional networks of palliative care providers are invited to choose a specific best practice from the pre-selected list and to submit a project proposal for an implementation trajectory. Each year, financial support is given by ZonMw for approximately 17 implementation trajectories. The grant applications are assessed by an independent quality working group. The criteria for granting this support include the requirement that the applicant must be a representative of the regional palliative care networks, the care organizations involved must reach a substantial number of patients with palliative care needs and the best practice chosen must be implemented within one year [10].

It is important to determine the effects of the National Quality Improvement Programme for Palliative Care, since a lot of professionals and financial resources are involved and ultimately patients and relatives must benefit from this great effort. Other large-scale improvement programmes have already been implemented and evaluated in other domains, e.g. regarding safety in health care and social care [11-15]. However, so far such large-scale programmes are a new feature within palliative care. Earlier evaluations in other health care domains not only focused on the effectiveness of the programmes but also described the implementation processes in order to enable an understanding of how the programmes take shape in practice, as well as of barriers and facilitators. Such a process evaluation can be referred to as opening the ‘black box’ of interventions [16], which is important in increasing the likelihood that interventions will continue to be used in practice after the research has finished. In palliative care too, it is important to bridge the gap between research and practice [17,18] and to maximize the likelihood of the long-term adoption of quality improvement projects in practice [19]. Therefore, it is important to describe both the effects and the process of this large-scale implementation of palliative care best practices.

### Objectives

This paper presents the study protocol of the evaluation study. The primary objective of this study is:

•To gain insight into the effects of the National Quality Improvement Programme for Palliative Care as a whole on the quality of palliative care at a national level.

Its secondary objectives are:

•To gain insight into the effects of the separate implementation trajectories of best practices on the quality of palliative care within the participating regional palliative care networks or organizations.

•To elucidate the measured effects by describing the implementation process and the barriers and facilitators within the separate implementation trajectories.

The specific research questions being addressed in the evaluation study are as follows:

1. What are the effects of the National Quality Improvement Programme for Palliative Care as a whole on the quality of palliative care? More specifically, what are the programme’s effects on:

a. The percentage of patients who die in their preferred place?

b. The extent to which patients and relatives feel in control regarding palliative care?

c. The extent to which patients and relatives experience well-coordinated palliative care?

d. The extent to which patients and relatives feel that the palliative care is concordant with their preferences and values and meets their needs in the physical, psychosocial and spiritual domains?

2. What are the effects of the separate implementation trajectories on the quality of palliative care at the level of the regional palliative care networks or care organizations involved? (with equivalent subquestions to questions 1a to 1d)

3. How and to what extent are the best practices implemented? More specifically:

a. Does the implementation of the best practices take place as planned?

b. What barriers and facilitators are there for the implementation of the best practices and what conditions have been created to ensure the best practices are maintained?

c. Are there any other factors or developments – inside or outside the participating organizations – that might have contributed to the measured effects of the programme as a whole or within the separate implementation trajectories?

## Methods

### Evaluation study design

The National Quality Improvement Programme can be considered as a complex intervention involving various implementation trajectories of various best practices within a continuously changing, uncontrolled environment. Therefore we opted for a quantitative effect evaluation with a pre-test post-test design (to answer research questions 1 and 2) in combination with a qualitative process evaluation with a post-test design (to answer research question 3). The combination of quantitative and qualitative methods is considered useful in gaining insight into the effects of the programme, as well as the implementation processes, barriers and facilitators for the implementation and internal or external factors that may influence the outcomes.

### Study population

**Figure 1:**
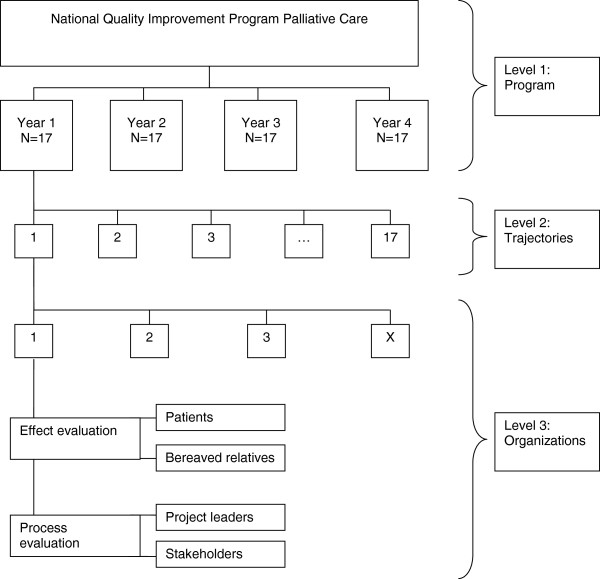
Organogram of the national quality improvement program palliative care.

All implementation trajectories receiving a grant in the National Quality Improvement Programme will participate in the evaluation study. Within the participating organizations, data will be collected from patients and bereaved relatives (see Figure 1). In implementation trajectories with more than five participating organizations, a purposive sample will be selected of care organizations with the highest number of patients meeting the inclusion criteria and representing all the different settings involved in the specific implementation trajectory.

An existent set of quality indicators measured among patients and bereaved relatives will be used for the effect evaluation [20]. The inclusion and exclusion criteria for patients and bereaved relatives are described in Additional files 2 and 3. Per trajectory we aim to include 15–20 patients and 30–40 bereaved relatives in each assessment period. As there are two assessment periods for the approximately 17 planned trajectories per year, this will result into a minimum of about 510 participating patients and 1020 participating bereaved relatives per year, which gives a total number of about 2040 patients and 4080 bereaved relatives over four years.

For the process evaluation, data will be collected among professionals. For each implementation trajectory, the project manager will be interviewed and a group interview will be done with other stakeholders involved. Assuming 17 implementation trajectories per year over four years, this will result in 68 interviews with project managers and 68 group interviews with stakeholders.

### Effect evaluation

**Figure 2:**
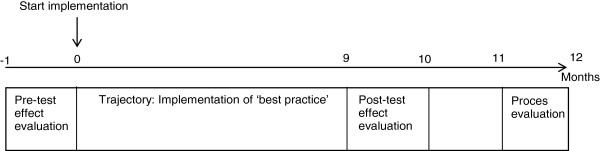
Chronological overview of one year of the program.

Pre-test and post-test measurements will take place in the effect evaluation, in month 0 and month 9 respectively (see Figure 2). During these two measurement months, eligible patients and bereaved relatives will be asked to participate.

#### Data collection

In the pre-test and post-test measurements, a set of quality indicators will be measured [20], making use of three types of measurement instruments:

•Numeric Rating Scales of six symptoms

•The Consumer Quality Index Palliative Care for patients

•The Consumer Quality Index Palliative Care for bereaved relatives

Numeric Rating Scales (NRS) will be used to measure the quality indicators ‘The % of patients with moderate to severe pain’, ‘The % of patients with moderate to severe fatigue’, ‘The % of patients with moderate to severe shortness of breath’, ‘The % of patients with moderate to severe constipation’, ‘The % of patients with moderate to severe anxiety’ and ‘The % of patients who feel moderately to severely depressed’. These indicators are associated with one specific programme objective, namely ‘Patients receive care for their needs in the physical and psychosocial domains’.

Patients will be reminded by one of their health care professionals to complete the NRS for the six symptoms (see Table 1) approximately the same time at three consecutive days. In principle, the patient will fill in the NRS scores. Only if the patient is not able to write will the health care professional assist the patient by writing down the NRS scores. The completed NRS forms will be collected by the health care professional after day 3 and send to the research team at the end of the measurement period.

**Table 1:** Numeric rating scales of 6 symptoms

**Symptoms (What number would you give your …symptom… on a scale of 0 to 10?**												
No pain	0	1	2	3	4	5	6	7	8	9	10	Much pain
No fatigue												Very fatigue
Not short of breath												Very short of breath
Not obstipated												Very obstipated
Not sad												Very sad
Not anxious												Very anxious

The Consumer Quality Index Palliative Care (CQ Index) is a validated questionnaire assessing quality indicators from the perspective of patients and bereaved relatives [21,22]. The quality indicators measured by this CQ Index are also directly related to the key objectives of the National Quality Improvement Programme (see Table 2). CQ index questions for instance concern patients’ or bereaved relatives’ experiences regarding the control over palliative care, the coordination of palliative care, the concordance between the care given and needs, preferences and values, and the physical, psychosocial and spiritual care received. In the version for bereaved relatives, questions are also asked about the actual and preferred place of death of the patient. The questionnaire version for patients consists of 56 items, while the version for bereaved relatives consists of 64 questions. Both questionnaire versions take approximately 30–45 minutes to complete. For inpatient patients, the questionnaire will be completed on the basis of an interview with a trained interviewer. Patients who receive care at home and bereaved relatives will receive the questionnaire by post to be completed and returned in a reply envelope to the research team. No reminders will be sent to non-responding patients, while a reminder will be sent after two weeks to non-responding bereaved relatives.

**Table 2:** Key objectives of the national quality improvement program and associated quality indicators, measured with the CQ index palliative care

**Key objectives of the programme**	**Quality indicators measured with CQ index version for patients**	**Quality indicators measured with CQ index version for bereaved relatives**
Patients die at their preferred place	-	*Percentage of patients who died in the location of their preference
		*Extent to which patients in the last month before their death were in the location of their preference
Patients and relatives feel they are in control regarding palliative care	*Extent to which patients experience respect for their autonomy	*Extent to which relatives indicate that the patient was asked about her/his opinions with regard to end-of-life decisions
	*Extent to which patients receive information from their caregivers about the expected course of the disease	*Extent to which direct relatives received information about the advantages and disadvantages of various types of treatment
	*Extent to which patients receive information about the advantages and disadvantages of various types of treatments	*Extent to which direct relatives received information that was understandable and unambiguous at the time of the patient’s death
	*Extent to which patients indicate that they receive understandable explanations	*Extent to which, according to the bereaved relatives, their autonomy was respected
		*Extent to which direct relatives were informed about the possibilities of aftercare
Patients and relatives see palliative care as being coordinated	*Extent to which patients know who the contact person is for the care	*Extent to which the bereaved relatives knew who the contact person was for the care
	*Extent to which patients experience expertise of caregivers and continuity of care	*Extent to which direct relatives perceived expertise of caregivers and continuity of care
	*Extent to which patients receive contradictory information	
Patients and relatives feel care to be concordant with their needs, preferences and values	*Extent to which patients are satisfied with “politeness” and “being taken seriously” by caregivers	*Extent to which the direct relatives felt that they were treated well in all respects by the caregivers
	*Extent to which patients experience respect for their privacy	*Extent to which direct relatives considered that the patient had the opportunity to be alone
	*Extent to which patients indicate that caregivers respect their life stance	*Extent to which the direct relatives had the opportunity to be alone with their relative
	*Percentage of patients who receive medical aids soon enough	
Patients and relatives receive care for their needs in the physical, psychosocial, and spiritual domains	*Extent to which patients receive support for their physical symptoms (pain, fatigue, shortness of breath and constipation)	*Extent to which relatives indicate that the patient had access to a counselor for spiritual problems
	*Extent to which patients receive help with physical care	*Extent to which relatives indicate that the patient received support with preparations for saying goodbye
	*Extent to which patients receive attention from their caregivers	*Extent to which relatives indicate that there was attention for the psychosocial and spiritual well-being of the patient
	*Extent to which patients receive support when they feel anxious or feel depressed	*Extent to which, according to the direct relatives, attention was paid to their own psychosocial and spiritual well-being
	*Extent to which patients indicate that they have access to a counsellor for spiritual problems	*Extent to which direct relatives felt supported by the caregivers immediately after the patient’s death
		*Extent to which a final conversation or discussion was held to evaluate the care and treatment

#### Data analysis

Descriptive statistics (frequencies, percentages and means) will be used to analyse background characteristics.

The effects of the separate implementation trajectories (research question 2) will be analysed using descriptive analyses as well as univariate analyses (with paired t tests or McNemar’s test). To assess the differences between pre-test and post-test measurements for the whole programme (research question 1), multilevel analysis will be used, including the levels of the participating care networks or care organizations, and the type of best practice. SPSS, STATA and ML-WIN will be used for the statistical analysis.

### Process evaluation

A qualitative process evaluation will be conducted nine to eleven months after the start of the implementation in a separate implementation trajectory (see Figure 2). The process evaluation will be used to explain the effects found (or not found) in the effect evaluation and provide insight into how the best practices are implemented.

#### Data collection

First, an analysis of relevant documents (e.g. implementation plans, and interim and final reports of the separate implementation trajectories) will provide insight into the implementation process. Second, we will conduct individual semi-structured interviews with the project managers of each trajectory. The topic list for the interviews will include questions on whether the best practice was implemented as planned, what the barriers and facilitators were for the implementation, what implementation strategies were used and whether the project managers felt implementation has been successful and effective. Third, for each implementation trajectory we will organize semi-structured group interviews with relevant other stakeholders (such as representatives of clients’ participation councils, nurses, doctors or other care professionals involved). A similar topic list as described for the individual interviews will be used for the group interviews. Both individual and group interviews will be audio-recorded.

#### Data analysis

The audio-recorded interviews will be transcribed verbatim. Subsequently a qualitative thematic analysis will be done, starting immediately after conducting and transcribing the individual and group interviews for a specific implementation trajectory. All relevant documents and interviews will be coded, analysed and discussed by at least two researchers. The coding process will be inductive, in the sense that themes and associated codes will emerge from the interview data through repeated comparison of relevant fragments within and between interviews. The coding process will be supported by the software package MAXQDA [23].

### Feedback and dissemination

The project manager and other stakeholders in a specific implementation trajectory will receive internal feedback reports on the results of the effect evaluation (after the pre-test measurements as well as after the post-test measurements) within two months after the measurement period. The prospect of getting feedback reports is important in order to increase the commitment of the project managers and stakeholders to participating in the measurements. In addition, the feedback reports will motivate the professionals involved to further improve the quality of their care, where needed.

Furthermore, interim reports about the progress of the programme as a whole will be published; these will also be made available to the general public. Also, additional international scientific publications about the effects and implementation of the programme are planned.

### Ethical considerations

The study protocol has been approved by the Medical Ethical Committee of the University Medical Center in Leiden, the Netherlands. Written informed consent of all patients will be obtained for the assessment of their symptoms and inpatients also consented with the Consumer Quality Index Palliative Care questionnaire, as it has to be completed by a trained interviewer. Patients who are at home and bereaved relatives received this questionnaire by post and for this no written informed consent is needed. Furthermore, in data collection and analyses procedures we follow the rules of Dutch Personal Data Protection Act (Wet Bescherming Persoonsgegevens). All personal identifiers will be removed or disguised in the analysis process to safeguard the privacy and anonymity of participants.

## Discussion

This evaluation study provides a unique chance to gain insight into the effects and implementation process of the National Quality Improvement Programme for Palliative Care and its separate implementation trajectories. The implementation of best practices within the framework of a long-term, broad nationwide programme can be considered as a complex intervention within a continuously changing, uncontrolled environment. Complex interventions are described by Pawson et al. as long journeys, embedded in multiple social systems, which are open and adaptive [24]. These characteristics also hold for the National Quality Improvement Programme for Palliative Care, which consists of the implementation of multiple best practices in different settings and with different starting points over a four-year period. Our programme is also relatively open and adaptive, for example as a result of the feedback reports and interim reporting. Feedback reports give the organizations insight into the current state of the quality of palliative care within their organization and might lead to additional actions to improve palliative care.

Randomised controlled designs (RCT) are often not applicable and feasible for the evaluation of complex interventions such as national quality improvement programmes [25,26]. When an RCT is not possible - as is the case in the evaluation of the National Quality Improvement Programme for Palliative Care - it is important to use a mixed-method design and to combine quantitative effect measurements with a qualitative process evaluation. Qualitative process evaluations, e.g. based on the qualitative analysis of interviews and documents, can help understand the effects (or sometimes the lack of effects) of interventions. Process evaluations are helpful in elucidating the implementation process and the interventions actually delivered (opening the ‘black box’) and in identifying other possible factors that may have contributed to the measured outcomes. One such factor may be an ‘investigation effect’ caused by the feedback reports. Therefore, during the process evaluation we will pay extra attention to possible additional actions of care organizations prompted by these feedback reports.

Evaluating complex interventions among patients with a limited life expectancy and among bereaved relatives is particularly challenging for researchers. In such populations, researchers have to take extra account of the burden of measurement for respondents, although many of them are willing to contribute to scientific research [27,28]. An additional challenge in our study in particular is the fact that palliative care patients are often 'scattered' throughout the organization or various teams or units. Due to the aim of the National Quality Improvement Programme for Palliative Care, i.e. to improve palliative care in general health care settings (like hospitals or home care), groups of patients with palliative care needs are less concentrated than in research within hospices, for instance, or specialist palliative care units. This means that an extra effort has to be made for the recruitment of patients and relatives as well as for informing all the professionals involved.

## Abbreviations

CQ Index: Consumer quality index; SPSS: Statistical package for the social sciences; ML-WIN: A software package for fitting multilevel models; MAXQDA: A software package for supporting qualitative data analysis.

## Competing interests

The authors declare that they have no competing interests.

## Authors’ contributions

NR, JH and AF conducted the study and drafted the manuscript. EN and LD participated in the design of the study and gave comments on drafts of the manuscript. All authors read and approved the final manuscript.

## Pre-publication history

The pre-publication history for this paper can be accessed here:

http://www.biomedcentral.com/1472-684X/13/5/prepub

## Supplementary Material

Additional file 1 Currently selected ‘best practices’. Click here for file

Additional file 2 Inclusion and exclusion criteria for patients. Click here for file

Additional file 3 Inclusion and exclusion criteria for bereaved relatives. Click here for file

## References

[B1] Seale C Changing patterns of death and dying Soc Sci Med 2000 51 917 930 10.1016/S0277-9536(00)00071-X 10972435

[B2] WHO http://www.who.int/entity/cancer/palliative/en/, visited November 6, 2013

[B3] IKNL Zorgpad stervensfase [Dutch care pathway for dying patients] 2013 Utrecht: IKNL

[B4] IKNL Signaleringsbox voor verzorgenden [signal box for nursing assistants] 2013 Utrecht: IKNL

[B5] Veerbeek L van der Heide A de Vogel-Voogt E de Bakker R van der Rijt CC Swart SJ van der Maas PJ van Zuylen L Using the LCP: bereaved relatives' assessments of communication and bereavement Am J Hosp Palliat Care 2008 25 207 214 10.1177/1049909108315515 18403578

[B6] Veerbeek L van Zuylen L Swart SJ van der Maas PJ de Vogel-Voogt E van der Rijt CC van der Heide A The effect of the Liverpool care pathway for the dying: a multi-centre study Palliat Med 2008 22 145 151 10.1177/0269216307087164 18372379

[B7] van der Heide A Veerbeek L Swart S van der Rijt C van der Maas PJ van Zuylen L End-of-life decision making for cancer patients in different clinical settings and the impact of the LCP J Pain Symptom Manage 2010 39 33 43 10.1016/j.jpainsymman.2009.05.018 19892509

[B8] Shaw KL Clifford C Thomas K Meehan H Review: improving end-of-life care: a critical review of the gold standards framework in primary care Palliat Med 2010 24 317 329 10.1177/0269216310362005 20156934

[B9] Burton CR Payne S Integrating palliative care within acute stroke services: developing a programme theory of patient and family needs, preferences and staff perspectives BMC Palliative Care 2012 11 22 10.1186/1472-684X-11-22 23140143 PMC3539873

[B10] ZonMw‘best practices’ of the Quality Improvement Programme for Palliative Care [Verbeterprogramma Palliatieve Zorg] http://www.goedevoorbeeldenpalliatievezorg.nl/, visited November 6, 2013

[B11] Ovretveit J Klazinga N Linking research to practice: the organisation and implementation of the netherlands health and social care improvement programmes Health Policy 2013 109 175 186 10.1016/j.healthpol.2012.11.005 23270882

[B12] Vos L Towards process-oriented care delivery in hospitals PhD thesis 2010 University Maastricht/NIVEL

[B13] Dückers M Changing hospital care: evaluation of a multi-layered organisational development and quality improvement programme Utrecht University/NIVEL 2009

[B14] Strating MM Nieboer AP Zuiderent-Jerak T Bal RA Creating effective quality-improvement collaboratives: a multiple case study BMJ Qual Saf 2011 20 4 344 50 10.1136/bmjqs.2010.047159 21270070 PMC3066797

[B15] Ovretveit J Klazinga N Meta-evaluation of the ten national quality improvement programmes in The Netherlands 2004–2009 2010 The Hague, the Netherlands: The Netherlands Organisation for Health Research and Development (ZonMw)

[B16] Broer T Nieboer AP Bal RA Opening the black box of quality improvement collaboratives: an actor-network theory approach BMC Health Serv Res 2010 10 265 10.1186/1472-6963-10-265 20825648 PMC2944272

[B17] Kutner JS A significant milestone for palliative care: imperative for dissemination and implementation research J Palliat Med 2011 14 1194 1195 10.1089/jpm.2011.9639 22050580

[B18] Grol R Improving the quality of medical care: building bridges among professional pride, payer profit, and patient satisfaction JAMA 2001 286 2578 2585 10.1001/jama.286.20.2578 11722272

[B19] Demiris G Oliver DP Capurro D Wittenberg-Lyles E Implementation science: implications for intervention research in hospice and palliative care The gerontologist 2013 [Epub ahead of print] 10.1093/geront/gnt022 PMC3954415 23558847

[B20] Claessen SJ Francke AL Belarbi HE Pasman HR van der Putten MJ Deliens L A new set of quality indicators for palliative care: process and results of the development trajectory J Pain Symptom Manage 2011 42 2 169 82 10.1016/j.jpainsymman.2010.10.267 21429703

[B21] Claessen SJJ Francke AF Deliens L Measuring patients' experiences with palliative care: the CQ-index palliative care BMJ Support Palliat Care 2012 2 367 372 10.1136/bmjspcare-2011-000055 24654223

[B22] Claessen SJ Francke AL Sixma HJ de Veer AJ Deliens L Measuring Relatives' perspectives on the quality of palliative care: the consumer quality index palliative care J Pain Symptom Manage 2013 45 5 875 84 10.1016/j.jpainsymman.2012.05.007 23017623

[B23] GmbH VERBI MAX QDA - a qualitative analysis software package 2013

[B24] Pawson R Greenhalgh T Harvey G Walshe K Realist review - a new method of systematic review designed for complex policy interventions J Health Serv Res Policy 2005 10 Suppl 1 21 34 10.1258/1355819054308530 16053581

[B25] Craig P Dieppe P MacIntyre S Michie S Nazareth I Petticrew M Developing and evaluation complex interventions: new guidance BMJ 2008 337 a1655 10.1136/bmj.a1655 18824488 PMC2769032

[B26] Campbell M Fitzpatrick R Haines A Kinmonth AL Sandercock P Spiegelhalter D Framework for design and evaluation of complex interventions to improve health BMJ 2000 321 694 696 10.1136/bmj.321.7262.694 10987780 PMC1118564

[B27] Gysels MH Evans C Higginson IJ Patient, caregiver, health professional and researcher views and experiences of participating in research at the end of life: a critical interpretive synthesis of the literature BMC Med Res Methodol 2012 12 123 10.1186/1471-2288-12-123 22900965 PMC3489694

[B28] Raijmakers NJ Clark JB van Zuylen L Allan SG van der Heide A Bereaved relatives' perspectives of the patient's oral intake towards the end of life: a qualitative study Palliat Med 2013 27 7 665 72 10.1177/0269216313477178 23442880

