# Supplementary material for: The effect and process evaluations of the national quality improvement programme for palliative care: the study protocol
Source: BMC Palliat Care. 2014 Feb 21;13:5. doi: 10.1186/1472-684X-13-5 (PMC3936932; doi:10.1186/1472-684X-13-5)
Supplement: Additional file 1 — Currently selected ‘best practices’. [file 1472-684X-13-5-S1.doc]

**Box 1: Currently selected ‘best practices’**

| - PaTz – a systematic approach to improve the quality and organization of care by timely identification of patients in need of palliative care and by drafting an advance care plan. (Dutch equivalent of the Golden Standard Framework) - Signal box for nursing assistants to timely identify palliative care needs in their patients - Dutch version of the Liverpool Care pathway for the Dying patient - STEM-inspirational cycle – a trajectory with professionals to accelerate expertise, to create awareness of the diversity of patients’ wishes and needs at the end of life, to improve communication-skills and to improve professionals’ ability to support patients and relatives at the end-of-life - Informare - a tailored method to provide timely information about palliative care to patients and relatives - Decision-making in palliative care – a decision tool for professionals to make decisions on end-of-life care by using clinical assessment for palliative care in a multidisciplinary team - Implementation of national guideline for Palliative Sedation in primary care - Advance Care Planning – a training for general practitioners to better recognize patients with palliative care needs in consultation with a specialist palliative care consultant - Utrecht Symptom Diary – training of using this tool systematically to evaluate the symptom burden of the patient and of adequately responding to the burden |
| --- |
