# Supplementary material for: The effect and process evaluations of the national quality improvement programme for palliative care: the study protocol
Source: BMC Palliat Care. 2014 Feb 21;13:5. doi: 10.1186/1472-684X-13-5 (PMC3936932; doi:10.1186/1472-684X-13-5)
Supplement: Additional file 2 — Inclusion and exclusion criteria for patients. [file 1472-684X-13-5-S2.doc]

**Box 2: Inclusion and exclusion criteria for patients**

Inclusion criteria for adult patients are:

- The patient has a life expectancy of less than 6 months, measured by the surprise question*, and/or undergoes palliative treatment, such as palliative chemotherapy, palliative radiotherapy, palliative surgery, or other treatments that aim to improve the quality of life and/or to extend life, but do not aim to cure the disease
- Is physically and mentally capable to respond to questionnaires and to understand Dutch.

Exclusion criteria for adult patients are;

- Comatose, deeply sedated, or dying patients
- Patients who have a care relationship shorter than one week

*Surprise question is: “Would I be surprised if this patient will to die within the next 6 months?” [1]

Reference

1. Murray S, Boyd K: Using the 'surprise question' can identify people with advanced heart failure and COPD who would benefit from a palliative care approach. Palliat Med 2011, 25:382
