# Supplementary material for: The effect and process evaluations of the national quality improvement programme for palliative care: the study protocol
Source: BMC Palliat Care. 2014 Feb 21;13:5. doi: 10.1186/1472-684X-13-5 (PMC3936932; doi:10.1186/1472-684X-13-5)
Supplement: Additional file 3 — Inclusion and exclusion criteria for bereaved relatives. [file 1472-684X-13-5-S3.doc]

**Box 3: Inclusion and exclusion criteria for bereaved relatives**

Inclusion criteria for bereaved relatives are;

- Has been a contact person (first contact person) of a deceased patient and has been involved in the care of the deceased patient who died after a sickbed
- The decease of the patient has been longer than 6 weeks ago and less than 6 months ago.

Exclusion criteria for bereaved relatives are;

- A contact person of a patient who died suddenly and unexpected.
